# Physicochemical and thermomechanical performances study for Timahdite sheep wool fibers application in the building's insulation

**DOI:** 10.1038/s41598-023-31516-9

**Published:** 2023-03-28

**Authors:** Aziza Atbir, Mhamed Taibi, Badr Aouan, Abdelhamid Khabbazi, Omar Ansari, Moha Cherkaoui, Toufik Cherradi

**Affiliations:** 1 GCC, Mohammadia School of Engineering, Mohammed V University in Rabat, EMI Rabat, Avenue Ibn Sina B.P. 765, Agdal, Rabat, Morocco; 2 Centre des Sciences des Matériaux, Laboratoire de Physico-Chimie des Matériaux Inorganiques et Organiques (LPCMIO), Ecole Normale Supérieure (E.N.S), Mohammed V University, Rabat, Morocco; 3 EMDD_CERNE2D, Mohammed V University in Rabat, EST Salé, 227 Avenue Prince Héritier, Salé, Morocco; 4 Energy Research Center, Thermal and Energy Research Team, ENSAM, Mohammed V University, Rabat, Morocco; 5 Laboratory of Applied Mathematics and Computer Science Decision, National Graduate Engineering School of Mines, Rabat, Morocco

**Keywords:** Ecology, Energy science and technology, Engineering, Materials science

## Abstract

The present research focuses on the development and thermomechanical characterization of unfired solid bricks based on clay (white and red) and Timahdite sheep wool, which are local, durable, abundant, and economical materials. As this clay material is incorporated with sheep wool in the form of yarn multi-layers in opposite directions. It achieves good thermal and mechanical performance and a lightness of these bricks as acquired progress. This new method of reinforcement offers significant thermo-mechanical performance for the composite for thermal insulation in sustainable buildings. Several physicochemical analyses to characterize the raw materials were used. Thermomechanical measurements to characterize the elaborated materials. The wool yarn effect was significant on the mechanical behavior of the developed materials at 90 days, with flexural strength from 18 to 56% for the white clay. And 8–29% for the red one. Decrease in compressive strength from 9 to 36% for the white clay and 5–18% for the red one. These mechanical performances are accompanied by thermal conductivity gain ranging from 4 to 41% for the white and 6–39% for the red for wool fractions: 6–27 g. This green multi-layered bricks from abundant local materials with optimal thermo-mechanical properties, qualified for the intended use for thermal insulation and energy efficiency in the construction and development of local economies.

## Introduction

Our global warming conditions require the use of renewable energies and commitment to the various international agreements on reducing greenhouse gases and different programs with a "Green Deal" orientation. The "eco-materials" to build "eco-housing" are the alternative to concrete, hence the importance of studying ecological composite materials that reduce this energy consumption. According to M’lahfi et al.^1^, energy consumption in buildings accounts for a significant share. The construction sector consumes up to 40% of total energy and contributes up to 30% of annual global greenhouse gas emissions; This causes real environmental damage. Morocco has committed itself to the Moroccan Thermal Construction Regulations to ensure energy-efficient buildings. Zong et al.^2^ stated that building walls with insulating materials saves energy and increases comfort levels. Casini et al.^3^ presented a work of buildings' insulating materials, examining their characteristics, costs, and environmental performances. Salih et al.^4^ presented work on fibers used as reinforcement in adobe bricks. They concluded that the properties of adobe bricks can be expected to improve with fibers addition. Asare and Danyuo^5^ studied the characterization of adobe materials reinforced with bamboo fibers. The compressive strength results showed an improvement in the reinforced bricks ranging from 7.2 MPa (at 5wt.% bamboo fiber) to 17.67 MPa (at 25wt.% fiber blocks). According to Rogovina et al.^6^, fibers derived from raw materials of animal origin are the second type of natural fibers used for composite reinforcement. Savio et al.^7^ presented experimental results of new thermal and acoustic insulation products, using waste as a matrix and other fibers waste as fillers. The results showed that panels have rigidity, workability, and satisfy thermal conductivity. Zach et al.^8^ are interested in alternative thermal insulation examination based on sheep wool. The thermal insulation was tested under various conditions, and its characteristics were close to those of conventional materials. Hegyi et al.^9^ investigated the mattresses' properties for water sorption–desorption competence in utmost moisture qualification. The analyses showed that these mattresses are thermal insulated materials environmentally friendly that have a slight effect on consumers' health. Streimikiene et al.^10^ declared that the lowest health impact is associated with, while alternatives such as recycled fibers, extruded polystyrene, and expanded polystyrene show the worst performance. Dénes^11^ et al. measured the properties used as a thermally insulating material. It was found that it could replace an extent hydrocarbon-based products. And still in the context of sustainability and eco-energy, Kunal et al.^12^ demonstrated the use of sheep wool fiber materials in the civil engineering sector. Due to the good quality of sheep wool fibers, it is recommended for insulating composites manufacture because of self-healing reinforcement, offering superior acoustic, thermal, and microscopic stiffness qualities and exhibiting high toughness, flexibility, and fracture-resistant structures.

Atbir et al. investigated the thermal behavior^13^ and conducted flexural tests on the samples at the 70-day term curing^14^ of a clay material reinforced by the wool grid layer method. They proved that this wool grid improves the thermomechanical properties of the biocomposite material. This work consists of another method of layer sequence in opposite directions of wool yarns, a different method from that previously published of the wool grid. And this is to improve the thermomechanical behavior and lightness of unfired bricks. The composite material developed (earth + wool yarns) is generally used for its thermal properties rather than its mechanical properties, but a minimum of mechanical strength is essential. So, it is subjected to stresses from its own weight and from the loads it carries. A building envelope made of this composite material must support at least its own weight. The determination of sufficient strength for a given use is therefore necessary for the optimal exploitation of the insulating and lightweight qualities. The content of the wool yarns, the mechanical strength, and the thermal conductivity are crucial parameters for determining the optimum point and choosing the right type of insulating building material. The thermal and mechanical properties of the developed material were studied, and the thermomechanically optimal composite was determined. This work aims to establish different experiments: physicochemical characterization by plasticity index, pH, infrared spectroscopy, x-ray diffraction of wool and clays, and the scanning electron microscopy of elaborated composites. Finally, the thermal properties, flexural, and compressive characteristics at set periods of 90-days were determined. This study is based on a project that aims to achieve the possibility of the good thermomechanical behavior of a durable composite from abundant local materials. These multi-layered composites are reinforced with animal yarns, bringing new perspectives for sustainable construction and thermal insulation. The physicochemical structure and innate thermal characteristics of wool fiber make it suitable as a reinforcing fiber for bio-composites. (Prevents thermal bridges, thanks to their surface scales, external tension differences cause the surface scales to open, which ensures resilience and prevents the fiber from settling over time. Wool fiber traps air in its hollow structure. It absorbs more than 33% of its weight in moisture due to its capillary structure). Clay is an insulating material that has always had the best qualities for thermal and acoustic building insulation. This article is divided as follows: the first section is devoted to the materials and methods; the second is to results and discussion. Finally, the third section is dedicated to the conclusion.

## Materials and methods

### Starting materials

#### Sheep wool

**Figure 1:**
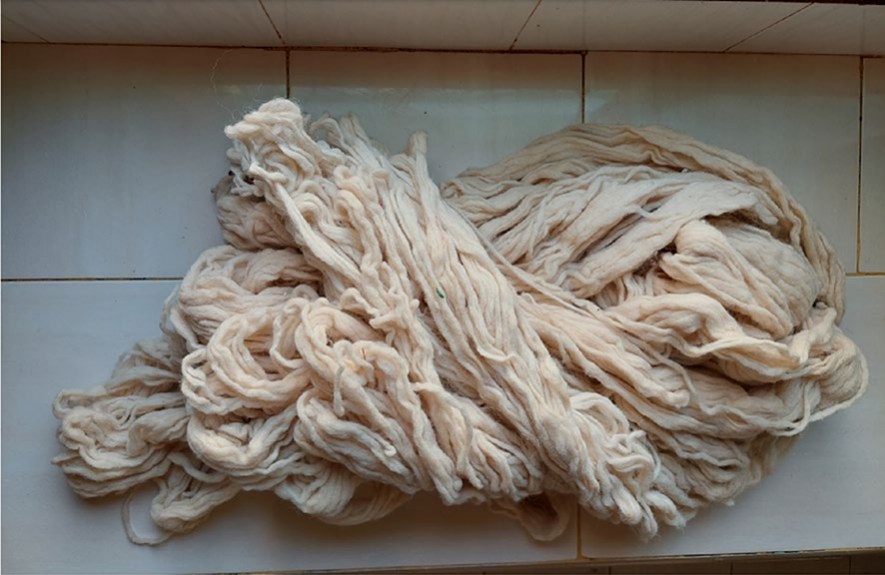
The yarn of sheep wool.

It is a dense fiber, hollow from the inside, curly and flexible, covering the sheep's skin. It is extracted by shearing once a year in Morocco in the Middle Atlas between May and June. Timahdit sheep wool was used in this work (Fig. 1).

#### Clays

**Figure 2:**
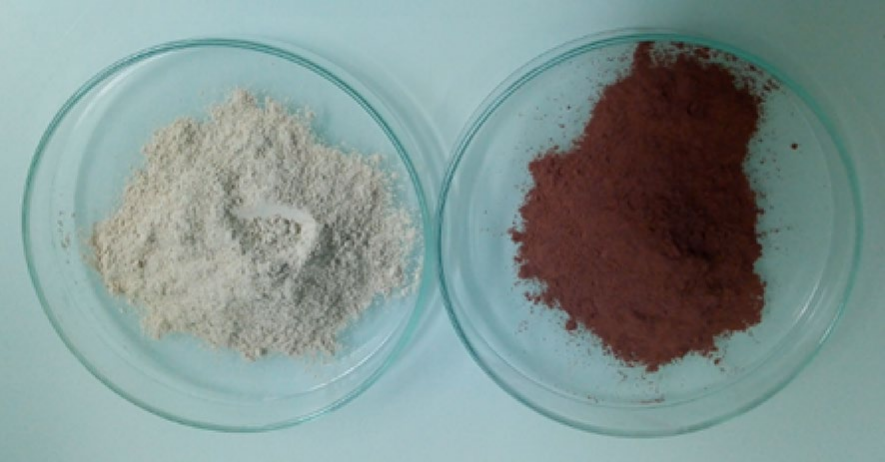
The white and red clay.

This study was carried out on the natural white (W) and red (R) clays from the Middle Atlas in Morocco (Fig. 2). The clay particle size is less than 0.008 mm.

### Insulating bricks 3D design

**Figure 3:**
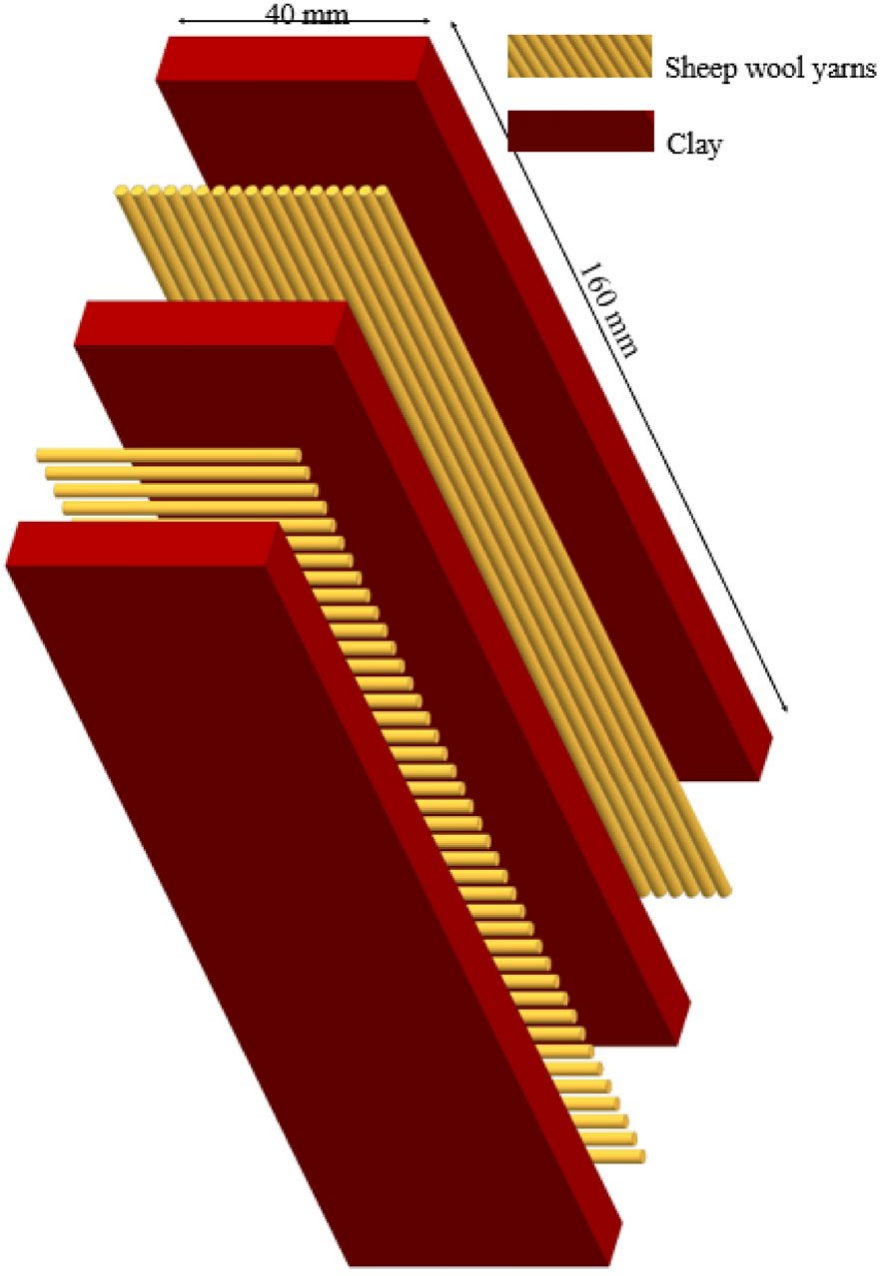
3D exploded design of a 40 mm thick insulating brick.

The 3D exploded design of durable insulating bricks was made before starting the practice (Fig. 3).

### Studied samples preparation

**Figure 4:**
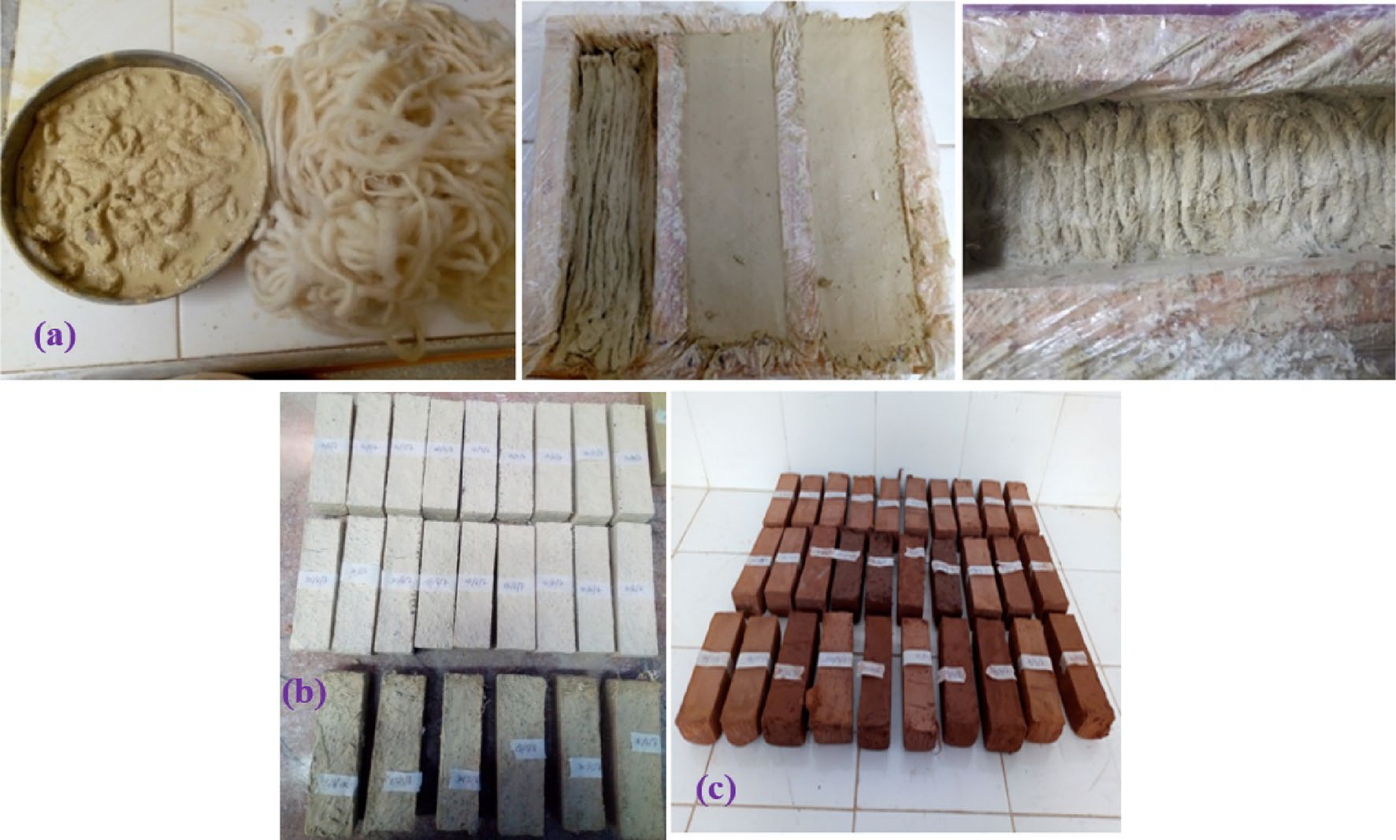
The prepared flexural samples (**a**), white category (**b**), red category (**c**).

Samples are prepared according to different mass fractions of wool yarns for each clay. The molds with volumes of 300.000 mm^3^ were used to manufacture samples for thermophysical tests, while the ones with volumes of 256.000 mm^3^, and 64.000 mm^3^ were manipulated respectively to elaborate specimens for flexural and compressive strengths tests. Based on the 30 mm thermal sample thickness, the maximum weight of yarns introduced experimentally is 27 g, which is equivalent to about four layers of wool yarn. The equivalent of the weight of this same yarn reaches 23 g for the flexural. And 5.75 g for the compressive samples according to their volume. Under 27 g, it was decided to experiment with different mass fractions of 18 g, 15 g, 6 g, and 0 g (Fig. 4). The mixing water absorption capacity for white clay is 37%, and that for red one is only 26% of their weight. The mixtures were well compacted with a manual press and small blows against the floor, as shown in Fig. 4. And this compaction energy is supplied manually. Such production is not a high cost; because of the low cost of sheep wool and the small quantities of wool yarn used in the preparation. And given the modern technology on the industrial level, this is easy to achieve.

Equations (1)–(3) were used to calculate the wool fraction ($v$), density (ρ), and composites-specific heat capacity ($C$) of elaborated composites according to mixture law.

1 $$v = \frac{{\rho_{{\left( {c - w} \right)}} - \rho_{c} }}{{\rho_{w} - \rho_{c} }}$$

2 $$\rho_{c - w} = v\rho_{w} + \left( {1 - v} \right)\rho_{c}$$

3 $$C_{c - w} = vC_{w} + \left( {1 - v} \right)C_{c}$$

With ρ_c-w_, ρ_c_ and ρ_w_ are the densities successively of the clay-wool, clay, and wool composites. C_c-w_, C_c_, and C_w_ are the specific capacities successively of the clay-wool, clay, and wool composites.

To study the thermal and mechanical performance of the produced material, it is necessary to conduct the possible physicochemical analyses of its raw materials, such as pH, Attereberg limit, IR spectroscopy, and X-ray diffraction, which allows us to know their properties and identify the compound composition. And Scanning Electron Microscopy to see the adhesion of the developed composite.

### Clays physicochemical characterizations

#### pH and Attereberg limit of clays

The pH and Attereberg limit analyses of clays were made in the Civil Engineering laboratory at the Higher School of Technology in Salé, Morocco. The clay's pH is determined according to the following procedure: putting the clay in the drying oven at 42 °C for two days, then placing 14 g of the dried clay in 50 mL of distilled water under agitation for one hour. And finally, letting it decantate for two hours. The Attereberg limit was calculated by the Casagrande device according to the NF P 94–051 standard to define the fine soil consistency and determine the plasticity index of the two clays.

#### IR spectroscopy characterizations

The functional groups of the clays and wool; were identified by Fourier Transform Infra-Red (FTIR) spectroscopy at the LPCMIO laboratory of the Higher Normal School in Rabat, Morocco. This analysis was performed at room temperature using a Bruker Platinum Attenuated Total Reflection (ATR) spectrometer with a resolution of 4 cm^-1^ and a scan number of 23 in the wavenumber range from 400 to 4000 cm^-1^.

#### X-ray diffraction of clays characterizations

The X-ray diffraction (XRD) analysis of white and red clays was also carried out at the Technical Support Units for Scientific Research of the CNRST in Rabat, Morocco, to identify the mineralogical composition of the studied materials. The XRD characterization of powder samples at room temperature is performed using the X'PERT PRO MPD device, which is equipped with CuKα radiation (λ = 1.54056 Å) and powered by a 3-kW generator with voltage ranging from 10 to 60 kV and current ranging from 5 to 80 mA. The readings were collected with a resolution of less than 0.04° in 2θ at a scan rate of 1°/min from 2θ = 3° to 90°. The diffraction spectra were treated by the X'Pert HighScore Plus software^15^, certified by Panalytical.

#### Scanning electron microscopy characterizations

The Scanning Electron Microscopy (SEM) characterization was carried out in the Technical Support Units for Scientific Research of the National Center for Scientific and Technical Research (UATRS-CNRST) in Rabat, Morocco. This characterization was realized using an apparatus called the FEI Quanta 450 FEG-focused ion beam system to visualize the morphology of the synthesized composites.

### Thermal characterizations

**Figure 5:**
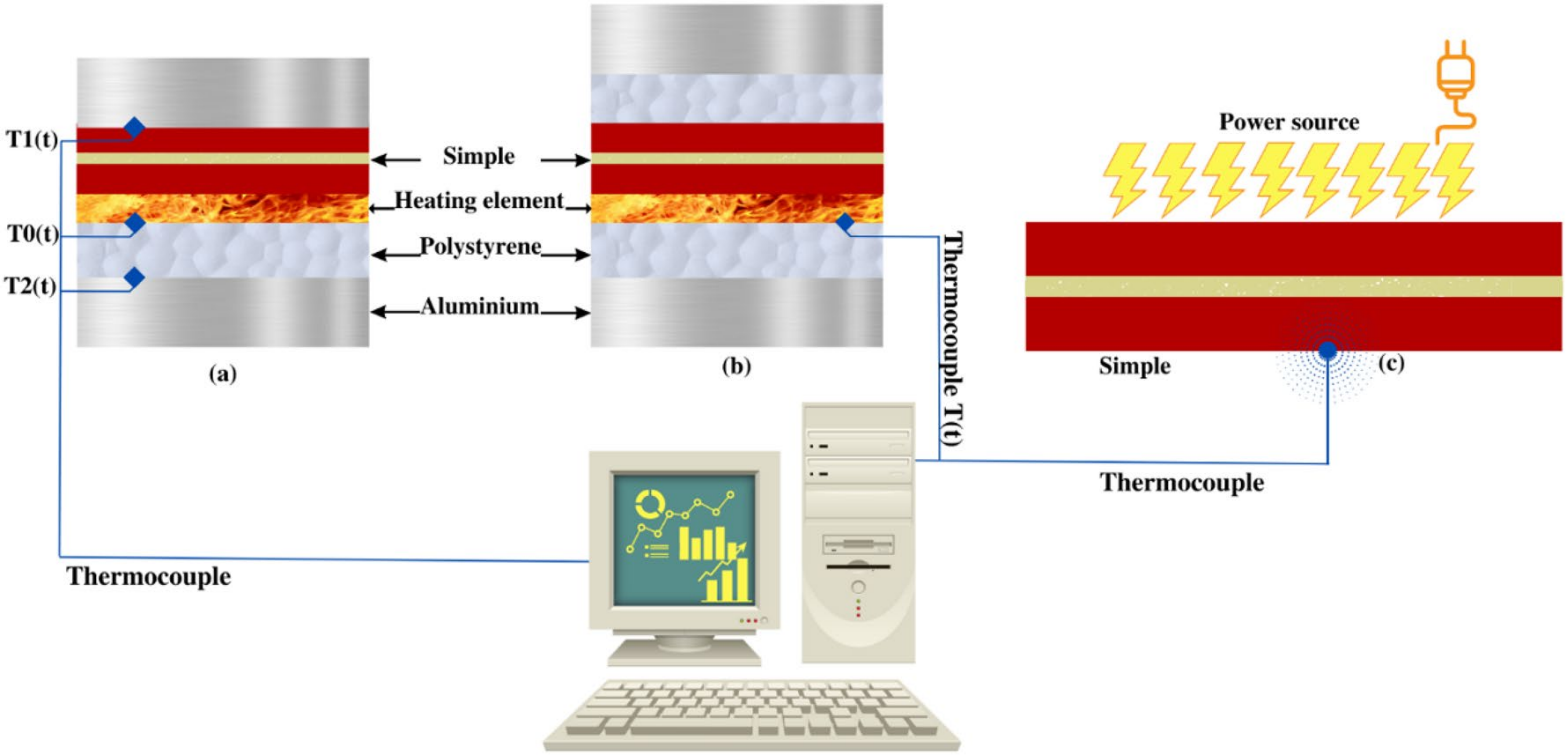
Schema of the thermal characterization methods principle.

The thermal conductivity of materials is performed using the asymmetric Hot Plate method (a) in a steady state. The thermal effusivity is determined by the Hot Plate method (b) in a transient state^16–19^. The Flash method (c) was used to determine the thermal diffusivity^19–21^. Figure 5 descript its principle. The thermal diffusivity and specific heat capacity can be estimated by the findings: thermal conductivity, thermal effusivity, and density of the developed material, using the relations (4) and (5). This characterization was also conducted at the Environment, Materials, and Durable Development laboratory at the Higher School of Technology of Salé, Morocco.

4 $$a = {\raise0.7ex\hbox{${\lambda^{2} }$} \!\mathord{\left/ {\vphantom {{\lambda^{2} } {E^{2} }}}\right.\kern-0pt} \!\lower0.7ex\hbox{${E^{2} }$}}$$

5 $$C = {\raise0.7ex\hbox{${E^{2} }$} \!\mathord{\left/ {\vphantom {{E^{2} } {\left( {\lambda \rho } \right)}}}\right.\kern-0pt} \!\lower0.7ex\hbox{${\left( {\lambda \rho } \right)}$}}$$

With: a (m^2^ s^-1^) thermal diffusivity, c (J kg^-1^ K^-1^) specific heat capacity, E (J m^-2^ K^-1^ s^-1/2^) thermal effusivity, λ (Wm^-1^ K^-1^) thermal conductivity and ρ (kg m^-3^) density.

### Mechanical characterizations

The sample's mechanical characterizations were studied by flexural and compressive strength using COMPACT-LINE CONTROLS EN 196-1 standard machine installed in the Environment, Materials, and Durable Development laboratory at the Higher School of Technology of Salé, Morocco. Load speed is equal to 50N/s for flexural and 500 N/s for compressive^19^.

### Dimensionless normalized coefficients of thermomechanical properties

To compare the thermal and mechanical parameters and to select the optimal thermo-mechanical sample, three dimensionless normalized coefficients: K_therm_ [Eq. (6)], K_flex_ [Eq. (7)], and K_comp_ [Eq. (8)], were inserted. The thermal resistance is inversely proportional to thermal conductivity, which increases with the increased fiber content. The wool introduced is accompanied by a decrease in compressive strength. Hence, the aim was to attain an optimum point equivalent to an excellent ratio of mechanical strength to thermal resistance^22^. K_therm_, K_flex_, and K_comp_ are defined as follows:

6 $$K_{therm } = \frac{{R_{ther measured} - R_{min} }}{{R_{max} - R_{min} }} = \frac{{1/\lambda_{therm measured} - 1/\lambda_{max} }}{{\frac{1}{{\lambda_{min} }} - \frac{1}{{\lambda_{max} }}}}$$

7 $$K_{flex} = \frac{{R_{flex measured} - R_{flex min} }}{{R_{flex max} - R_{flex min} }}$$

8 $$K_{comp } = \frac{{R_{comp measured} - R_{comp min} }}{{R_{comp max} - R_{comp min} }}$$

With:

K_therm_ is the dimensionless standardized parameter of thermal resistance. R_therm measured_ is the thermal resistance of a sample, and λ_therm measured_ is the thermal conductivity measured for each sample. R_max_ and R_min_ are respectively the maximum and minimum thermal resistance found. λ_max_ and λ_min_ are respectively the corresponding maximum and minimum thermal conductivity. K_flex_ is the dimensionless standardized parameter of flexural strength. R_flex measured_ is the flexural strength measured for each sample. R_flex max_ and R_flex min_ are respectively the maximum and minimum flexural strengths found. K_comp_ is the dimensionless standardized parameter of compressive strength. R_comp measured_ is the compressive strength measured for each sample. R_comp max_ and R_comp min_ are respectively the maximum and minimum compressive strength found.

### Ethical statements

This article does not contain any studies with human participants or animals performed by any of the authors.

## Results and discussions

### Clays physicochemical Characterization results

#### Clays pH

**Figure 6:**
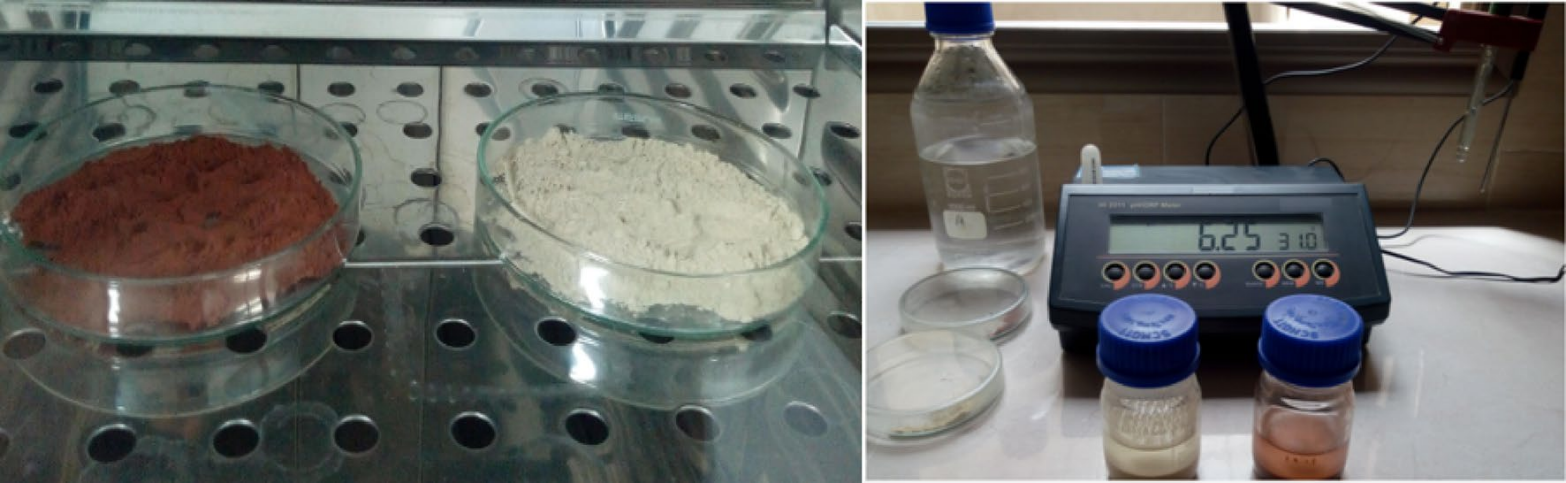
The clays pH.

The pH analyses obtained of the two clays are as follows (Fig. 6):pH _White clay_ = 7.12 Neutral, thus it is ideal soil.pH _Red clay_ = 8.16 Basic, thus it is alkaline earth.

#### Attereberg limit

The analysis results are as follows, plasticity index (I_P_) = liquidity limit (W_L_)—plasticity limit (W_P_).

According to Table 1, the I_P_ difference between W_L_ and W_P_ is equal to 14.69 for the white clay, which is a medium plasticity soil, and I_P_ = 8.10 for the red clay, which is a low plasticity soil.

**Table 1:** The plasticity index of clays C_B_ and C_R_.

Samples	C_W_	C_R_
W_L_	38.78	24.04
Wp	24.09	15.94
I_P_	14.69	8.10

#### Infrared spectroscopy characterization results

##### Infrared spectrum of white and red clays

**Figure 7:**
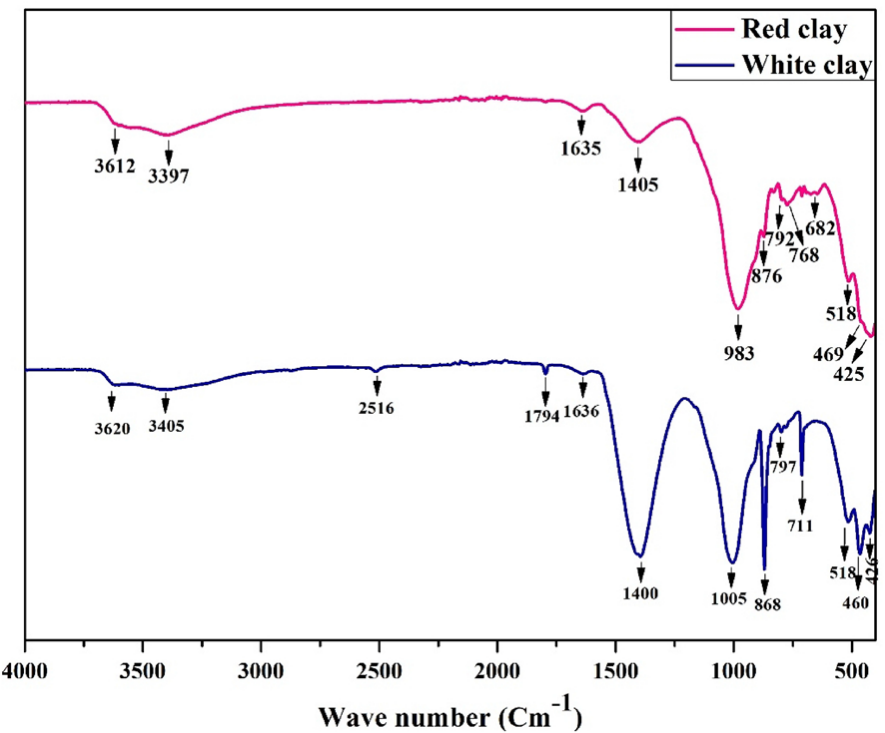
Infrared spectrum of the Red and white clays.

Figure 7 presents the natural clay's FTIR spectra. Table 2 lists the band's attributions. The absorption bands around 3620 and 3405 cm^-1^ in the white clay are attributed to the asymmetric and symmetric OH vibration groups^23^. The absorption bands located at 3612 and 3397 cm^-1^ in the red clay are assigned to the asymmetrical and symmetrical stretching vibrations of structural OH groups^24^. The characteristic bands around 2516 and 1794 cm^-1^ in the white clay are attributed to the carbon dioxide (CO_2_) vibration absorbed by the mineral clay^25^. The band observed at 1636 cm^-1^ in the white clay is characterized by HOH vibration groups ascribed to H_2_O absorbed from the atmosphere^26,27^. An absorption peak at 1635 cm^-1^ in the red clay is ascribed to the HOH functional grouping, which enters the formation of water molecules constituting the clay structure^28^. The two peaks close to 1405 and 876 cm^−1^ in red clay correspond to the C=O vibration in carbonates^26,29^. The band close to 1400 cm^-1^ is associated with C=O vibrations in calcite Ca (CO_3_)^30^. This band appears more intense in the white clay spectrum compared to the red one, which explains the high content of calcite present in white clay. The bands observed at 1005 and 426 cm^-1^ in the white clay, which are characteristic of Si–O stretching vibrations, appear more intense than those located at 983 and 425 cm^-1^ in the red clay, which are also specific for Si–O bonds^29,31^. The peak at 868 cm^-1^ in the white clay is caused by the C=O vibration of inorganic carbonate^32^. The bands observed at 797 and 711 cm^-1^ in the white clay are attributed to the Si–O–Al vibration group and confirm the occurrence of mineral quartz in this natural clay^33^.

**Table 2:** Frequency in cm^-1^ recorded on the white and red clay attributed to the molecular grouping.

Clay type	Bands (cm^-1^)	Assignments
White	3620 and 3405	Asymmetric and symmetric stretching vibrations of OH
Red	3612 and 3397	
White	2516 and 1794	Stretching vibration of CO_2_
White	1636	Functional grouping of H–O–H
Red	1635	
Red	1405 and 876	Bending vibrations of –C=O in carbonates
White	1400	
White	1005 and 426	Stretching vibrations of Si–O–T (T = Si or Al) bonds
Red	983 and 425	
White	868	Bending vibrations of C=O in carbonates
White	797 and 711	Stretching and Bending of Si–O–Al bonds
Red	792 and 768	
Red	682	Bending vibrations of Si–O–Si bonds
White	518	Bending vibration of Al–O–Si bonds
Red	518	
White	469	Bending vibration of Si–O-Si bonds
Red	460	

The existence of two consecutive peaks at 792 and 768 cm^-1^ in the red clay are the results of the Si–O–Al deformation vibrations, which seem less dense than the two previous ones that existed in the white clay^34^. The one band at 682 cm^-1^ in the red clay is related to the Si–O–Si vibrations of quartz^35^. The band close to 518 cm^-1^ in the white clay indicates the Al–O–Si bond elongation^36^. The bands at 518 cm^-1^ are owing to Al–O–Si vibrations in the red clay^26^. The peak at 469 cm^-1^ is related to Si–O–Si vibrations from the SiO4 tetrahedra in the white clay^37^. The band located at 460 cm^-1^ in the red clay attributed to Si–O–Si also looks less intense than its predecessor, one found in the white clay^26^.

##### Infrared spectrum of wool

**Figure 8:**
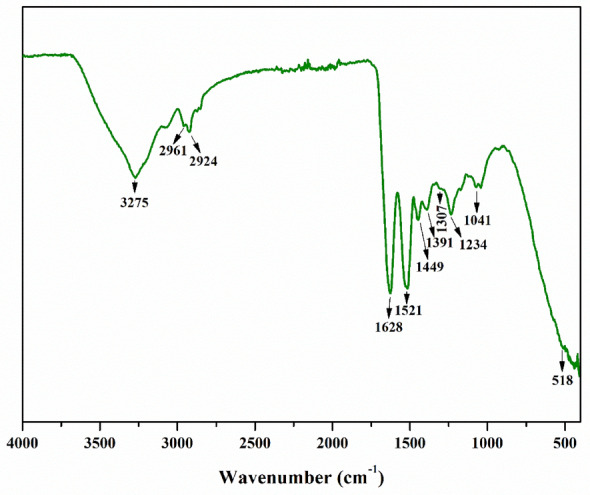
Infrared spectrum of wool.

Figure 8 shows the FTIR spectra of the natural sheep wool. Table 3 lists the typical bands assigned to peptide links (–CONH). Jiang et al.^38^ studied wool by dissolving amino acids and cleavage of disulfide bonds, which was ascertained using spectroscopy (Raman, FTIR, NMR), XRD, XPS, and amino acid analysis. The results showed that the protein macromolecular backbone and secondary structure of surface-dissolved wool are not lost by oxidation acid. Pakkaner et al.^39^ studied the water-soluble keratose proteins obtained from Ovis Aries wool. The wool samples and the extracted keratose proteins were characterized using FTIR, XRD, SEM, and TGA techniques. The peak observed at 3275 cm^-1^ is attributed to the vibration of the (-NH_2_) amino groups (amide A)^40^. The bands at 2961 and 2924 cm^-1^ are associated with asymmetric stretching vibrations of the CH_3_ and CH_2_ functional groups, respectively^41,42^. The peak near 1628 cm^-1^ is assigned to the O–H vibration mode due to bound water (amide i)^43^. Amid band is noted at 1521 cm^-1^ and is ascribed to both N–H in-plane and C-N vibrations (amide ii). The absorption band at 1449 cm^-1^ is related to delta CH_2_ symmetric vibrations^44^. The absorption at 1391 and 1307 cm^-1^ are linked respectively to the oscillations of the C-H and C-O bonds. The peak observed at 1234 cm^-1^ is due to the vibrations of C-N and C-O (amide iii)^40^. The band around 1041 cm^-1^ is ascribed to vibration [C–O–C] in the ester group^45^. The peak at 518 cm^-1^ represents the bending vibration of the N–H group^45^.

**Table 3:** Frequency in cm^-1^ found on attributed to the molecular grouping.

Bands (cm^-1^)	Assignments
3275	–NH_2_ stretching vibration
2961	CH_3_ asymmetric stretching
2924	CH_2_ asymmetric stretching
1628	O–H bending mode due to bound water
1521	C–N stretching and N–H in-plane bending vibrations
1449	Delta CH_2_ symmetric bending
1391	C–H bending
1307	C–O flexural vibration
1234	C–O and C–N stretching vibrations
1041	Stretching of –[C–O–C]– ester group
518	NH bending vibration

According to the data, the wool infrared spectrum corresponds to Zein which is a protein. Some behavior aspects of regenerated protein are satisfactory or comparable to those of natural wool protein.

#### X-ray diffraction of clays characterization results

**Figure 9:**
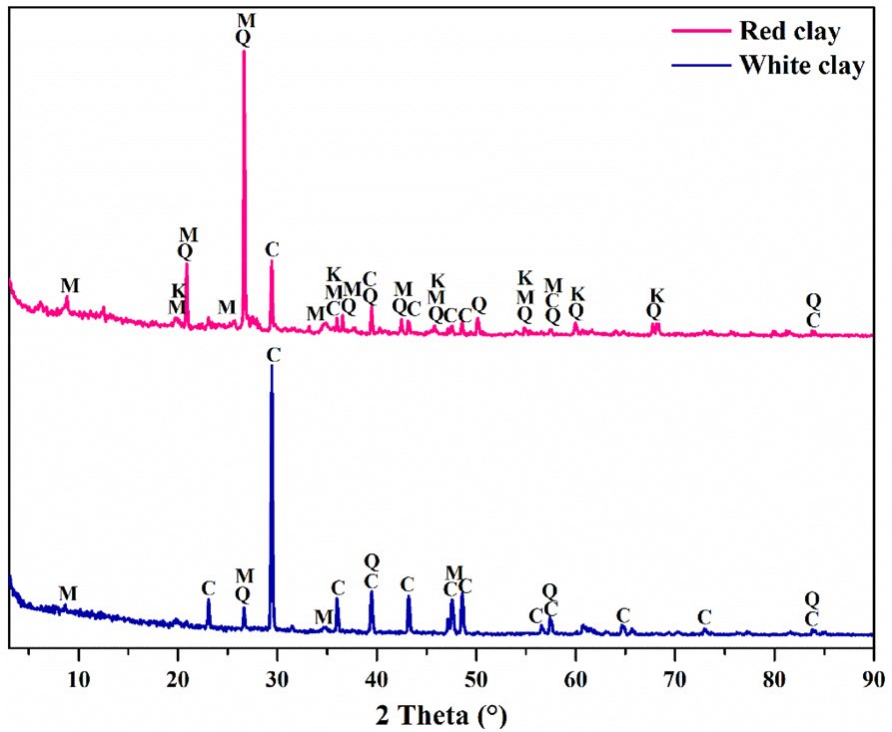
XRD pattern of the white and red clays. (K = Kaolinite, Q = Quartz, C = Calcite, M = Muscovite).

Figure 9 shows the XRD results for the mineral compositions of the white and red clay samples. The diffractogram of the natural white clay revealed the presence of distinct peaks for both quartz and calcite as major mineral phases and other peaks differentiating muscovite as a minor phase. It indicates that the white clay consists predominantly of quartz and calcite crystals, with a small quantity of muscovite. The DRX pattern of the natural red clay also shows distinct peaks for the same mineral phases found in the white clay pattern, with the appearance of kaolinite phase peaks. This observation revealed that red clay has a mineralogical composition principally consisting of quartz and muscovite, with a lesser amount of calcite and kaolinite. Moreover, the characteristic peak of calcite at around 29.40° (2θ) appears more intense in the XRD pattern of white clay, while the quartz peak at 26.50° (2θ) appears more intense in the XRD pattern of red clay. That shows that white clay contains a significant amount of lime, which explains its color, unlike red clay, whose color is attributed to the presence of a certain amount of kaolinite. According to these findings, it was concluded that both clays possess a crystalline structure with a somewhat different mineralogical composition. Which can be explained by the different formation properties. The comparison of the two chemical analysis methods, infrared spectroscopy, and X-ray diffraction, confirms that the FTIR data agrees with the XRD analysis.

#### SEM of manufactured composite characterization results

**Figure 10:**
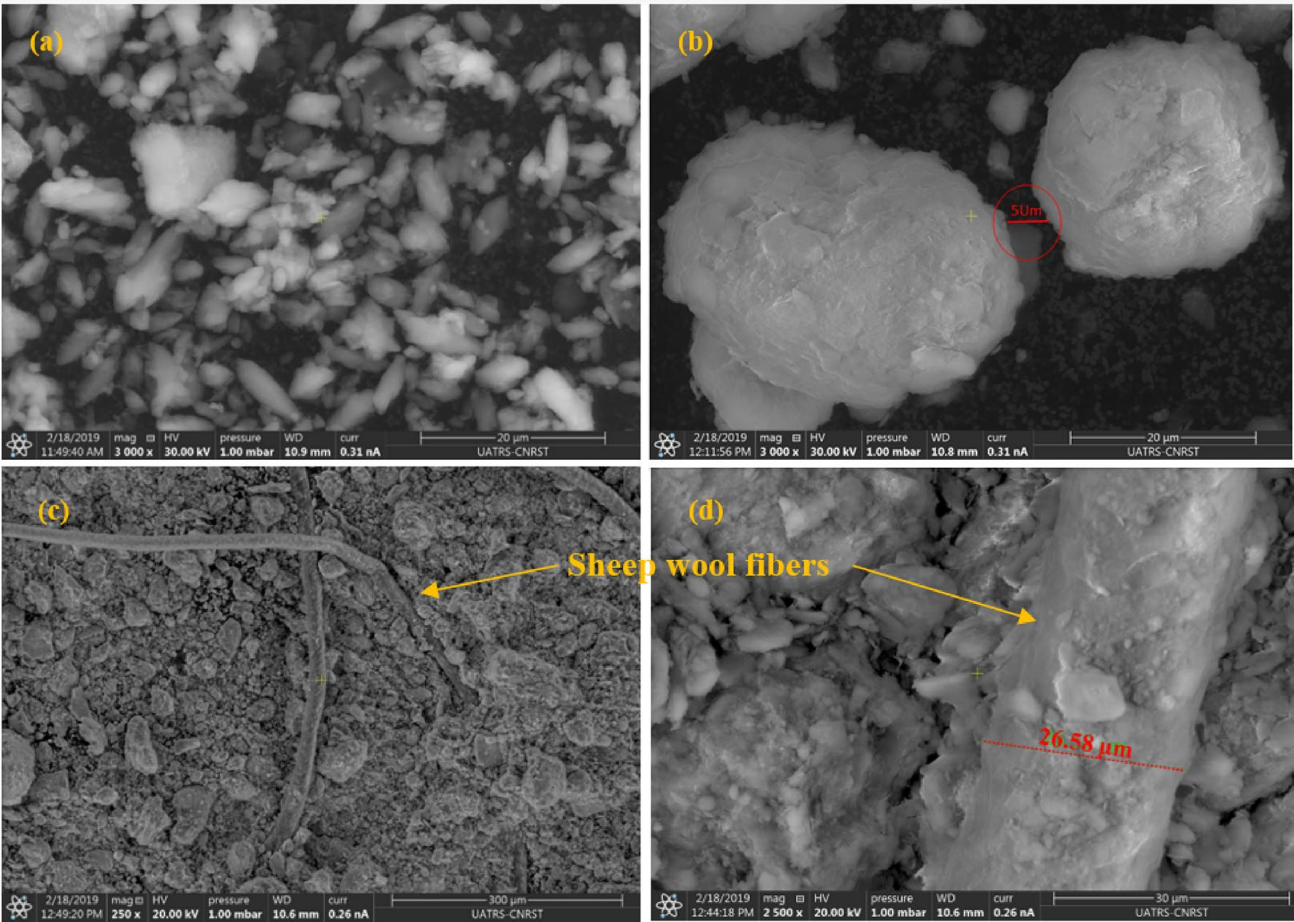
SEM picture of clay-wool composite material.

Figure 10 shows the SEM picture of the manufactured material. Photo (a) illustrates the white clay particles, which have a longitudinal shape, magnified ×3000, with a scale of 20 μm. And (b) the red clay particles, with a diameter of 5 µm between two particles. These red particles have a round shape magnified ×3000, with a 20 μm scale. Photos (c and d) illustrate the sheep wool fibers impregnated into the clay matrix and show good adhesion between the two components. (c) shows the sheep wool fibers that are naturally hollow at 250× magnification with a 300 μm scale. (d), shows the fiber diameter of 26.58 μm at 2500 × magnification with a 30 μm scale. This Fig. 10 shows the successful adhesion between the sheep wool fibers in the clay matrix.

### Thermal characterizations results

#### Samples density

**Figure 11:**
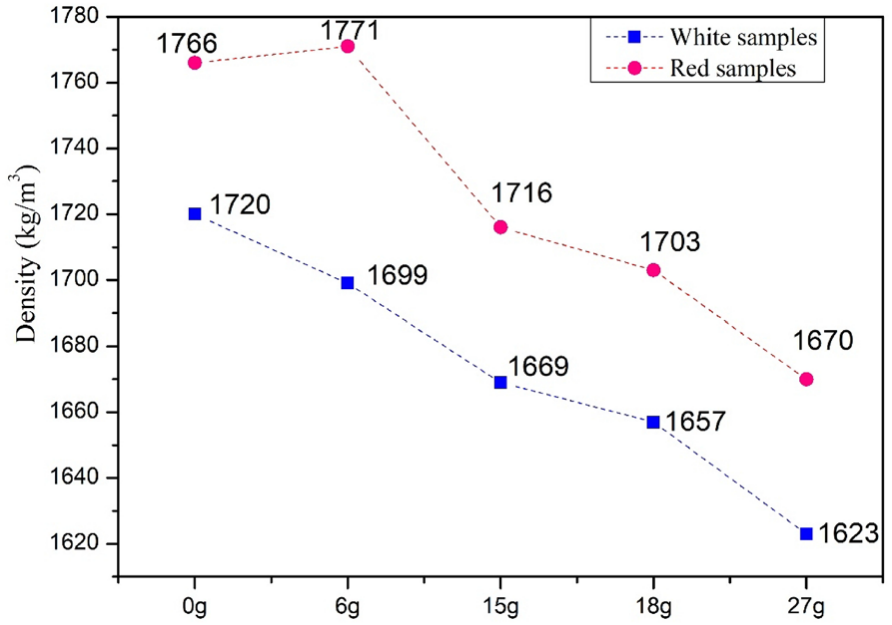
The density of white and red samples by sheep wool fibers content.

As shown in Fig. 11, incorporating different sheep wool mass fractions in the clay matrix decreased the sample's density. There is an increase in the samples' wool fraction, which decreases their density for the two categories. That can be explained by the porosity increase in multilayer composites. The natural hollow structure of sheep wool is more appreciated when used of a large fraction in the mix. Improve the composite lightness of the 1720–1623 kg/m^3^ for 0 g fraction of sheep wool (control sample) compared to 27 g fraction for white categories; and 1766–1670 kg/m^3^ for 0–27 g for red. It was noted that the masses values of white composites are lower than red composites. The mixing water absorption capacity shows that the percentage of water used for the white clay is 37%, and the red requires only 26%. After the evaporation of absorbed water, white clay is lighter than red clay.

#### Thermal conductivity, Thermal effusivity, and diffusivity

**Figure 12:**
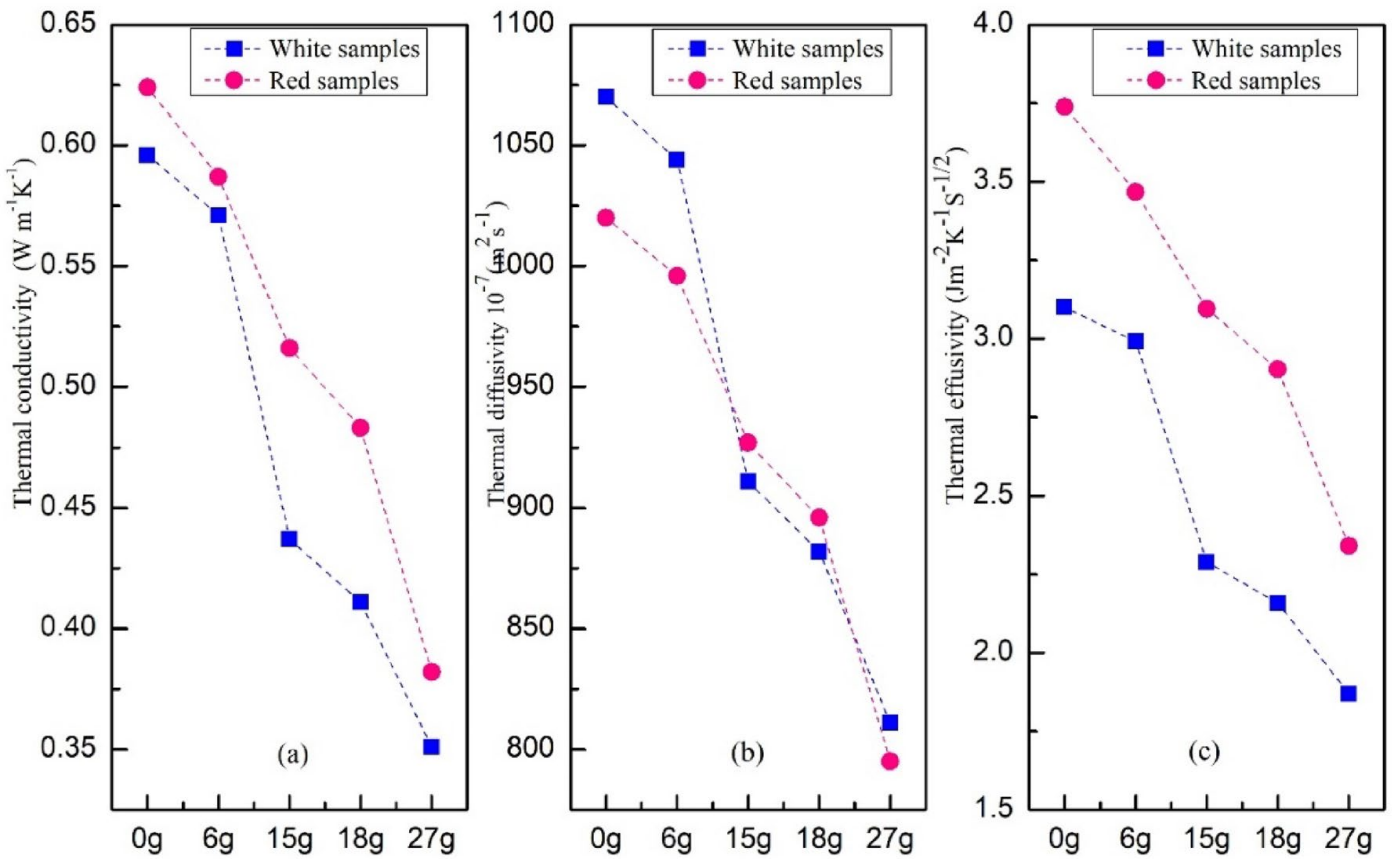
(**a**) Thermal conductivity, (**b**) thermal diffusivity, and (**c**) thermal effusivity of all samples. **Gain = 100*(λ _clay pure_ − λ _sample_)/ λ _clay pure_.

Figure 12 presents the thermal behavior of conductivity “λ”, effusivity “E”, and diffusivity “a” as a function of the wool fraction in the prepared samples. It was seen that the thermal conductivity of brick decreases by adding wool fibers for two composite categories. This decrease is significant for the 27 g sample that reaches λ_W_ = 0.351 W/m.K and λ_R_ = 0.382 W/m.K, compared to the 0 g sample that is λ_W_ = 0.596 W/m.K and λ_R_ = 0.624 W/m.K. The thermal conductivity behavior is due to the porosity increases in the clay composite with sheep wool increasing percentage. Decreasing bricks' thermal conductivity. That means the manufactured material is less favorable to heat transfer. It also noticed that the thermal conductivity values of white samples are lower than the red ones, which means white clay is less conducive to heat transfer. The λ gain varies from 4 to 41% for white and 6–39% for red for the wool fractions 6–27 g.

Thermal effusivity is the parameter characterizing absorption and energy storage. Thermal effusivity is dependent on thermal conductivity and capacity. The higher the thermal ability of the insulation material, the more it prevents the stored heat from contributing to the temperature rise, which means low-temperature contact between the surrounding medium and this manufactured material. It noted that the thermal effusivity decreases with wool fibers addition for two composite categories. That means that the porosity increases as the wool fiber fraction increases, and the thermal effusivity decreases. Thus, thermal effusivity is inversely related to porosity. This decrease is significant in the sample case 27 g that reaches E_B_ = 811 Jm^-2^ K^-1^ s^-1/2^ and E_R_ = 795 Jm^-2^ K^-1^ s^-1/2^ compared to the sample 0 g that achieves E_B_ = 1070 Jm^-2^ K^-1^ s^-1/2^ and E_R_ = 1020 Jm^-2^ K^-1^ s^-1/2^. The E gain varies from 2 to 24% for white and 2% to 22% for red for the wool fractions 6–27 g.

The results show a decrease in the estimated thermal diffusivity according to an increase in the wool yarn fraction for two composite categories. These results mean that increasing the wool percentage in the mixture acts positively under the porosity effect on the thermal diffusivity. This decrease reaches a_W_ = 1.870.10^-7^m^2^s^-1^ and a_R_ = 2.341.10^-7^m^2^s^-1^ for the 27 g sample compared to a_W_ = 3.100. 10^-7^m^2^s^-1^ and a_R_ = 3.738. 10^-7^m^2^s^-1^ for the 0 g sample. Decreasing the estimated thermal diffusivity that delays heat propagation means little heat exchange within this insulating material. And it was noticed that the thermal diffusivity values of white samples are lower than the red ones, which means white samples have a lower exchange capacity than red. The gain of a varies from 4 to 39% for white and 7–37% for red for the wool fractions 6–27 g. In this study, the thermal analysis showed a significant effect of layers of sheep wool yarn on the thermal behavior of the composites produced. The thermal performance of the white categories' is lower than the red ones. This work agrees with the other studies^13,46–50^ (Table 4) that demonstrated the decreasing thermal properties after fiber reinforcement.

**Table 4:** Comparison of the literature studies thermal performance.

References	Types of additives and fibers studied	Thermal performance
^13^	Investigated the thermal behavior of a clay material reinforced by the wool grid layer	The triple λ (W/m K), E (J m^-2^ K^-1^ s^-1/2^), and a (10^-7^m^2^ s^-1^) varies between (0.6, 873, 3.70) for 100 clay to (0.42, 751, 3.45) for clay + 15 g of sheep wool
^46^	Studied the thermal conductivity of three clay types of Morocco	The pairs λ (W/m K) and a (10^-7^m^2^ s^-1^) equal to (0.350, 3.50) for Tamansort clay, (0.368, 3.68) for Essaouira clay, and (0.457, 4.57) for Marrakech clay
^47^	Studied the composed material clay, water, and sisal fibers by considering two mass fractions: 2% and 4%	The pairs λ (W/m K) and a (10^-7^m^2^ s^-1^) equal to (0.649, 3.368) for pure clay decreased to (0.576, 3.116) for 4% of sisal fibers
^48^	Studied the thermal conductivity of clay-straw composite material	The pairs λ (W/m K) and a (10^-7^m^2^ s^-1^) varies between (0.504, 3.728) for 100 clay and (0.263, 2.858) for clay + 5% straw
^49^	Studied the thermal performance of unfired sand bricks, silt, and clay for construction in the north of France	The obtained λ is 0.9 W/m K. E is 936,49 Jm^-2^ K^-1^ s^-1/2^. a is 9.23.10^-7^m^2^/s for these bricks
^50^	Studied the thermal conductivity of clay bricks reinforced by three ecological materials: olive waste (OW)-clay, date palm fibers (DPF)-clay, and Straw-clay	The quadruple λ (W/m K), E (W s^1/2^ .m^-2^ K^-1^), a (Wm^-1^ K^-1^), and % (of ecological materials) obtained are: (0.40, 715.28, 3.15: 30) to (0.55, 904.07, 3.75:10) for OW-clay (0.28, 625.78, 2.01: 30) to (0.45, 811.23, 3.08:10) for DPF-clay (0.26, 510.02, 2.69: 30) to (0.43, 763.86, 3.20: 10) for straw-clay Compared to (0.65, 1001.12, 4.21) for pure clay

### Mechanical characterizations results

**Figure 13:**
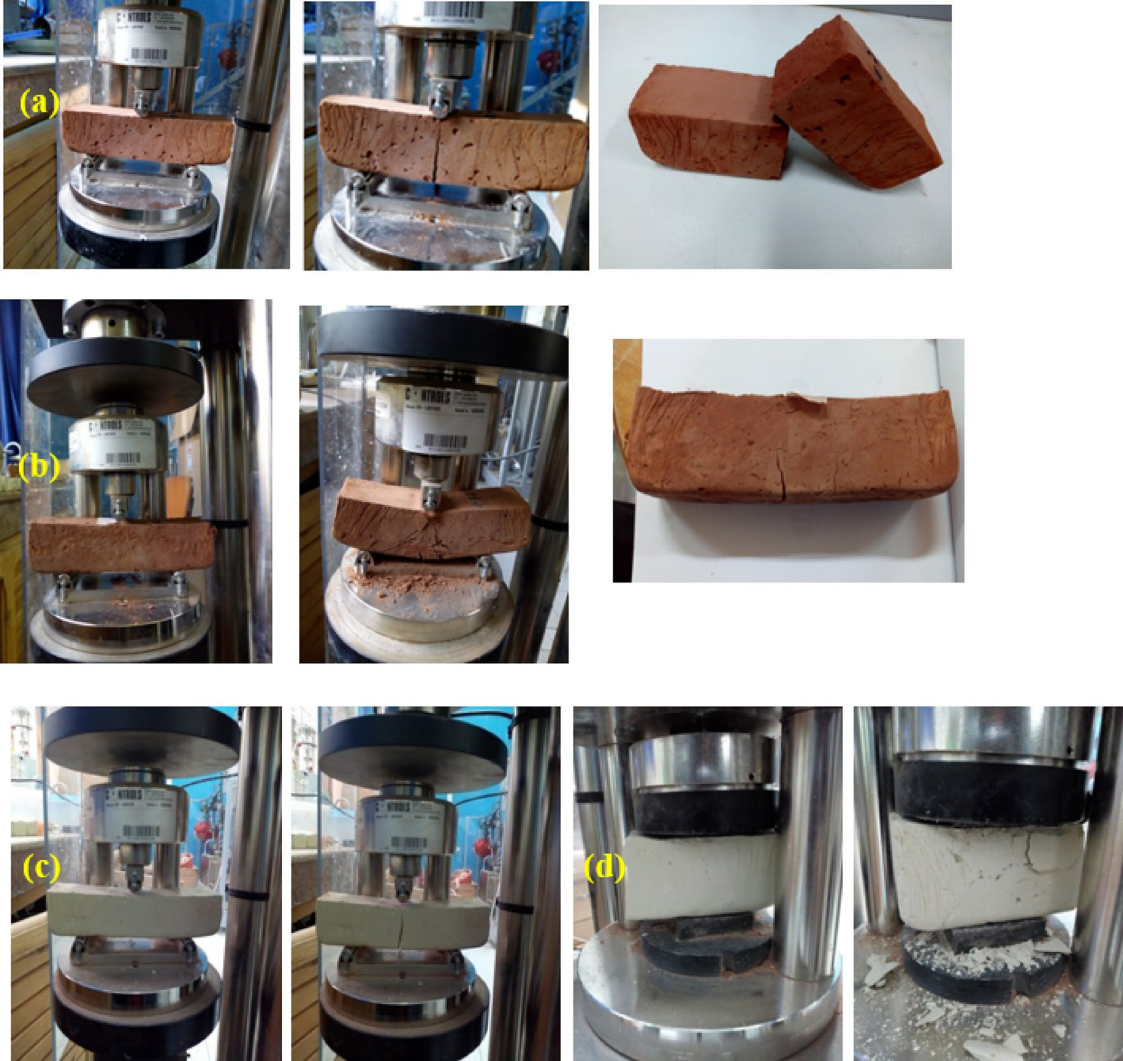
White and red clay samples before and after destruction by flexural and compression.

A certain resistance level is necessary for optimal insulation quality exploitation. Thus, to know the mechanical answers at 90 days that can be applied to the elaborated samples, let us limit ourselves to the resistance to flexural (a, b and c) and compression (d) (Fig. 13). These tests and study periods were chosen to see if there would be remarkable results according to different mass fractions of wool yarns and aim to find a starting point for a good setting and behavior.

(a) Shows the pure clay samples, which press a complete fraction after bending destruction. (b) shows the samples reinforced with layers in opposite directions of wool yarns, which press a non-complete crack. (c) illustrates the white clay pure samples after destruction by bending and (d) after destruction by compression.

**Figure 14:**
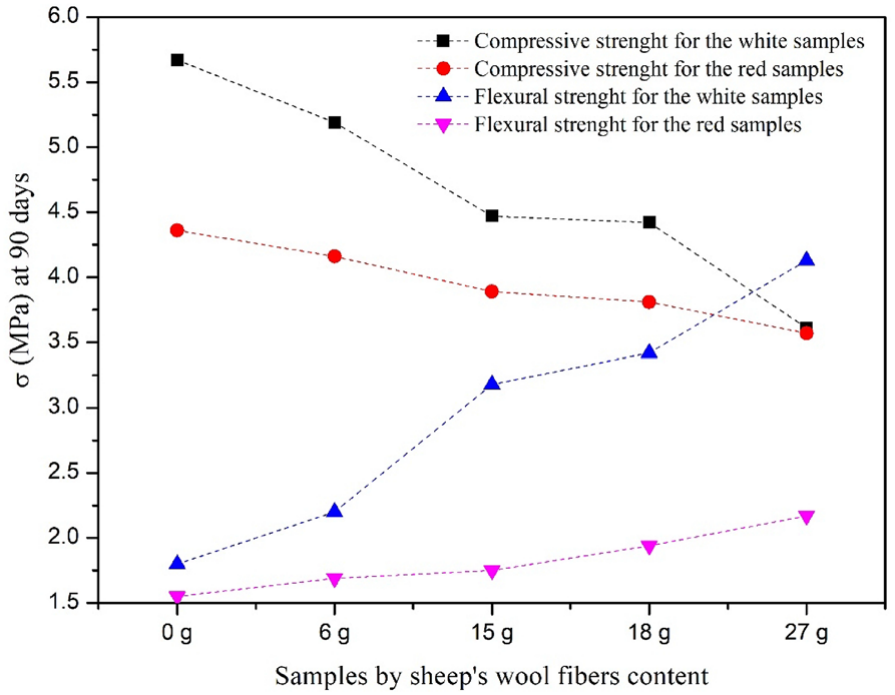
Mechanical behavior at setting periods 90 days of all samples. **Gain = 100*(ϭ _clay pure_ − ϭ _sample_)/ ϭ _clay pure_.

From Fig. 14, the results show that the wool fibers addition affects the mechanical strength. The compression decreases, and the flexural increases for both developed categories at 90 days. In this study, the physicochemical results indicate that: density, the wool fibers adhesion in the clay matrix, and preparation method affect the composite mechanical properties. The layers form in opposite directions of wool yarn increase in solid bricks leads to flexural strength increase. This experimental result is explained by the high resistance of the wool yarns to the flexural stress due to their flexibility and good adhesion between the clay and this type of opposite direction layers, which present a remarkable hardness of the composite, and maintain it against the phenomenon of shrinkage that causes cracks in the clay composites during drying. So, increasing the wool yarn content in the composite improves flexural strength. The flexural gain varies from 18 to 56% for white and 8–29% for red for wool fractions 6–27 g compared to the pure clay. The 27 g is 56% higher for white than 6 g and 29% for red.

The increase in wool yarn layers in solid bricks leads to a decrease in compressive strength. The compressive lower varies from 9 to 36% for white and 5–18% for red for wool fractions 6–27 g compared to the pure clay. The 27 g is 36% lower for white than 6 g and 18% for red. And this decrease in compressive strength, compared to the initial material, is due to the porous nature of the wool fibers, which leads to a decrease in the density of the composites as a function of the wool content and a decrease consequently in compressive strength. These bricks, reinforced by layers in opposite directions of wool yarns, do not show the phenomenon of shrinkage. Sample 6 g is strong in compression and weak in bending. Sample 27 g is strong in bending and weak in compression. The white clay's flexural and compressive strength is higher than the red. This behavior is consistent with previous work^14,51–56^ (Table 5). This study examined the mechanical behavior of composite materials reinforced and stabilized with durable fibers. The results conclude that the additive fibers with this method improve flexural strength and do not affect the compressive strength much.

**Table 5:** Comparison of the literature studies mechanical performance.

References	Types of additives and fibers studied	Mechanical performance
^14^	Investigated the compression behavior at a 70-day setting period of a clay material reinforced by the wool grid layer method	The finding indicated that ϭ comp = 2.05 MPa for 0L (100 clay), to ϭ comp = 4.38 MPa for 4.5L (clay + 15 g of sheep wool)
^51^	Study the clay fabric variation with its mechanical behavior and physical properties, Scanning Electron Microscopy (SEM) has been widely used	Results showed that the soil microstructure influences the mechanical behavior of geomaterials
^52^	Studied the using local materials in construction. An evaluation of the lime’s stabilizing clay at six different contents (0%, 10%, 20%, 30%, 40%, 50%, and 70%)	The thermophysical results showed that the stabilized clay blocks show optimal mechanical performance compared to the reference samples
^53^	Presented the mechanical properties results of a model earth-based reinforced with a fiber sisal natural and synthetic polypropylene	The results showed that the fiber reinforcements modified the composite microstructure. This strengthening occurred via an elastic stress transfer mechanism attributed to fiber-matrix interactions
^54^	Studied the needles using the possibility of three types of pine as reinforcing fiber in the adobes manufacture, compared to using wheat straw	The results conclude that pine needles improve the strength by up to 24% compared to straw use, the compressive strength of adobes made with pine needle fibers are 3.2 MPa Pinus halepensis (pn1), 3.3 MPa pinea (pn2), and 2.4 MPa pinaster (pn3), compared to 2.7 MPa for straw adobe
^55^	Evaluated the addition of wood chips from pruned vine shoots as a manufacturing additive at various percentages	The authors concluded that the 10% maximum of wood chip content is limited by the compressive strength values and the water absorption
^56^	Examined the mechanical behavior of composite materials improved by the effect of ecological and sustainable fibers additives	It was observed that adding fibers to the composite does not mainly affect much the compressive strength. Moreover, even if the sample is damaged, it maintains its coherence and does not present a great deformation

### Dimensionless normalized coefficients of thermomechanical properties results

**Figure 15:**
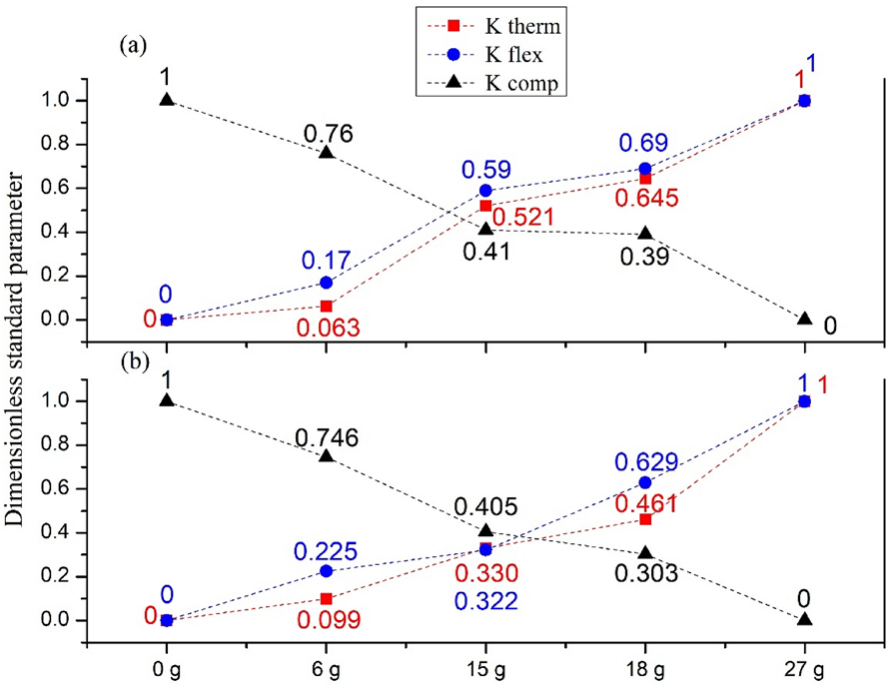
Standardized parameters depending on fiber fraction of all samples (**a**) White, (**b**) Red.

Figure 15 indicates the three parameters K_therm_, K_flex_ and K_comp_ development.

As shown in Fig. 15, the intersection of the thermal resistance graphs K_therm_, K_flex_, and the compressive strength graph K_com_ is within the 13 g content for the white samples (a). And is around 15.5 g for the red (b). Therefore, the 15 g wool-reinforced composite is thermomechanically optimal for both types of clay. This research was mainly limited to the thermomechanical characterization, given the destination of the new eco-material to the thermal insulation in the construction field. Thus, an optimum thermomechanical point was determined, at which there is a gain on the thermal side and not much loss on the mechanical side.

The optimal fraction of wool 15 g was chosen for both clay types. This choice is justified by the following reasons:

- The 15 g fraction has good thermal resistance with the highest Mechanical strength/Thermal resistance ratio,
- The minimum weight of the wool layers in the mix keeps the best mechanical resistance.

## Conclusion

This article focuses on eco-construction and thermal insulation for sustainable energy efficiency. Depending on the thermomechanical characterization, we have shown that layers in opposite directions of wool yarns improve flexural strength and does not affect the compressive strength much at 90 days of clay material compared to the pure clay composite. Provided that the amount of wool yarns does not exceed 15 g for both composite categories.

- 27% in conductivity, 15% in effusivity, and 26% in diffusivity for the white.
- 17% in conductivity, 9% in effusivity, and 17% in diffusivity for the red.
- 43% gain in flexural strength and a 21% decrease in compressive strength for the white.
- 11% gain in flexural strength and an 11% decrease in compressive strength for the red.

The wool yarns positively affect the studied composite thermal and flexural behavior and slightly reduce the compressive strength values. Therefore, the experimental study confirms the expectations of having green multi-layered bricks from abundant local materials with optimal thermo-mechanical properties, qualified for the intended use, and radically advances thermal insulation and energy efficiency in the construction and development of local economies. In this research, the 15 g wool-reinforced composite is recommended for a dynamic thermal simulation study on the heating and cooling consumption in a building and an economic study for the energy bill as a future perspective of this research.

## Data Availability

All data generated or analyzed during this study are included in this published article.

## Abbreviations

0 g: Sample with 0 g of yarns (0 Layers)
6 g: Sample with 6 g of yarns (1 Layer)
15 g: Sample with 15 g of yarns (2 Layers)
18 g: Sample with 18 g of yarns (3 Layers)
27 g: Sample with 27 g of yarns (4 Layers)
M: Masses (kg)
ρ: Density (kg m^-3^)
E: Effectiveness (J m^-2^ K^-1^ s^-1/2^)
λ: Thermal conductivity (W m^-1^ K^-1^)
a·10^–^^7^: Diffusivity (m^2^ s^-1^)
C: Specific heat capacity (J m^-3^ K^-1^)
σ: Resistance (MPa)
L * W * H: Length * Width * Height (mm^3^)
